# LINC01013 Is a Determinant of Fibroblast Activation and Encodes a Novel Fibroblast-Activating Micropeptide

**DOI:** 10.1007/s12265-022-10288-z

**Published:** 2022-06-27

**Authors:** N. M. Quaife, S. Chothani, J. F. Schulz, E. L. Lindberg, K. Vanezis, E. Adami, K. O’Fee, J. Greiner, M. Litviňuková, S. van Heesch, N. Whiffin, N. Hubner, S. Schafer, O. Rackham, S. A. Cook, P. J. R. Barton

**Affiliations:** 1 National Heart and Lung Institute, Imperial College London, London, UK; 2 MRC London Institute of Medical Sciences, London, UK; 3 Program in Cardiovascular and Metabolic Disorders, Duke-National University of Singapore, Singapore 169857, Singapore; 4 Max-Delbrück-Center for Molecular Medicine in the Helmholtz Association (MDC), Berlin, Germany; 5 DZHK (German Center for Cardiovascular Research), partner site Berlin, Berlin, Germany; 6 Princess Máxima Center for Pediatric Oncology, Utrecht, The Netherlands; 7 Cardiovascular Research Centre, Royal Brompton and Harefield Hospitals, Guy’s and St Thomas NHS Foundation Trust, London, UK; 8 Wellcome Centre for Human Genetics, University of Oxford, Oxford, UK; 9 Berlin Institute of Health at Charité – Universitätsmedizin Berlin, Berlin, Germany; 10 National Heart Centre Singapore, Singapore, Singapore

**Keywords:** Myocardial fibrosis, lncRNA, Micropeptide, Small open reading frame, Translation

## Abstract

Myocardial fibrosis confers an almost threefold mortality risk in heart disease. There are no prognostic therapies and novel therapeutic targets are needed. Many thousands of unannotated small open reading frames (smORFs) have been identified across the genome with potential to produce micropeptides (< 100 amino acids). We sought to investigate the role of smORFs in myocardial fibroblast activation.

Analysis of human cardiac atrial fibroblasts (HCFs) stimulated with profibrotic TGFβ1 using RNA sequencing (RNA-Seq) and ribosome profiling (Ribo-Seq) identified long intergenic non-coding RNA LINC01013 as TGFβ1 responsive and containing an actively translated smORF. Knockdown of LINC01013 using siRNA reduced expression of profibrotic markers at baseline and blunted their response to TGFβ1. In contrast, overexpression of a codon-optimised smORF invoked a profibrotic response comparable to that seen with TGFβ1 treatment, whilst FLAG-tagged peptide associated with the mitochondria. Together, these data support a novel LINC01013 smORF micropeptide-mediated mechanism of fibroblast activation.

## Introduction

Myocardial fibrosis is a common final pathology of almost all forms of heart disease. It leads to arrhythmia and haemodynamic failure, and is an independent predictor of mortality [1–3]. No current treatment for myocardial fibrosis has been shown to offer prognostic benefit [4]. Although some major determinants of myocardial fibrosis are recognised, inhibition of upstream molecules, such as transforming growth factor beta-1 (TGFβ1) has detrimental pleiotropic effects, limiting their viability as therapeutic targets [5, 6]. Therefore, identification of novel therapeutic targets, downstream of TGFβ1, is urgently needed.

Cardiac fibroblasts comprise a heterogeneous mesenchymal cell population that produce and remodel extracellular matrix, and whose activation is a precursor to the fibrotic response [7]. The final consequence of fibroblast activation is their transdifferentiation into extracellular matrix (ECM) producing and contractile myofibroblasts, the persistence of which turns the fibrotic response from physiological to maladaptive, with the production of pathological quantities of extracellular matrix. Markers of fibroblast activation include interleukin-11 *(IL11),* as a key determinant of fibroblast activation [8], snail *(SNAI1),* which is necessary for myofibroblast transdifferentiation and fibrosis [9–13], and periostin *(POSTN)* and alpha-smooth muscle actin (α-SMA, *ACTA2)* which are both established markers of myofibroblast transdifferentiation [7, 14].

Small open reading frames (smORFs) are increasingly recognised to play a significant role in regulating cellular processes [15, 16]. These short regions with coding potential can be identified both within the upstream and downstream regions of messenger RNAs traditionally labelled as untranslated (UTRs), and within long non-coding (LNC) RNAs—an abundant class of RNAs with largely undescribed functional roles [17]. In order to visualise smORFs which are actively translated, ribosome profiling (Ribo-Seq) leverages the property of ribosomes to protect RNA from endonuclease digestion. Sequencing and mapping of ribosome-protected fragments (RPFs) of RNAs generate a snapshot of regions of active translation [18–20]. From these studies, it is now clear that many regions, previously annotated as non-coding, in fact contain smORFs which are not only actively translated but can be demonstrated to produce biologically active micropeptides with a diverse range of actions, including in the cardiovascular system [15, 16, 21–25].

LINC01013 is a largely uncharacterised intergenic lncRNA, previously considered to be non-coding, which has been seen to have a role in chondrogenesis [26], cancer progression [27, 28] and regulation of DNA repair in endothelial cells [29]. A recent epigenomics study found it to be co-ordinately expressed with a key fibrotic gene *CCN2/CTGF* [30] in the context of human umbilical cord vascular endothelial (HUVEC) cells, suggesting a potential role in fibrosis. To date, no study has addressed the possibility of LINC01013 encoding a bioactive peptide.

Here, we demonstrate a direct role of LINC01013 in the response of cardiac fibroblasts to TGFβ1. Moreover, we use ribosome profiling to identify a small open reading frame (LINC01013ORF) with strong evidence of translation. LINC01013ORF is independently able to activate cardiac fibroblasts and encodes a small peptide that localises to the mitochondria. Taken together, these data show that LINC01013 and its encoded micropeptide are likely to play a key role in fibroblast activation and represent novel targets for antifibrotic therapy.

## Methods

### Cell Culture of HCF Cells

Human atrial cardiac fibroblasts (passage 1) for in vitro analyses were obtained commercially from iBiologics (lines 1125, 1153, 1156, 1162, 1164, 1166). Culture medium for all lines was Dulbecco’s modified eagle medium (DMEM, Gibco), with 10% foetal bovine serum (Gibco) and 1% penicillin/streptomycin (Gibco). Cells were maintained at 37° with 95% air and 5% CO_2_. Tissue culture plates were pre-treated with poly-D-lysine (2 μg/cm^2^, Sigma). Cells were passaged by trypsinisation with 0.05% trypsin (Thermofisher) and centrifuged at 1000 rpm for 5′, before resuspension, counting and re-plating at appropriate concentration.

### Cell Culture of HeLa Cells

Human HeLa cells (ATCC) were cultivated in a humidified incubator at 37 °C with 5% CO_2_ using Dulbecco’s modified eagle medium (DMEM) with high glucose (4.5 g/l), 10% foetal bovine serum (FBS), 2 mM L-glutamine and 1 mM sodium pyruvate. Medium was changed after 48 h and cells were split at confluency rates of 80-90% using standard trypsinization techniques.

### RNA-Seq and Ribo-Seq

Ribo-Seq and RNA-Seq datasets were obtained using human cardiac fibroblasts treated with TGFβ1 as previously described in [31]. The adapters were trimmed using Trimmomatic V0.36 [32] and reads that aligned to mitochondrial RNA, ribosomal RNA and transfer RNA using Bowtie [33] were discarded as previously described in [31]. The remaining reads were aligned to the human genome (hg38) using STAR [34] and read coverage on known coding genes was calculated using featureCounts from the Subread package [35]. Differential expression analysis was carried out using DESeq2. Ribotaper [36] was used to detect actively translated small open reading frames (smORFs) based on Ribo-Seq and RNA-Seq reads.

### siRNA Knockdown

HCFs (iBiologics) were seeded at 2.4 × 10^5^ per well of 6 W plates. After 24 h, cells were washed twice with OptiMEM (Thermofisher) and transfected with pooled anti-LINC01013 or nontargeting control siRNAs (Dharmacon) using Lipofectamine RNAiMax (Invitrogen) following manufacturer’s protocols and to a final siRNA concentration of 100 nM for 24 h. Transfection media was then changed to serum-free DMEM + / – 5 ng/ml TGFβ1 (Bio-Rad).

### qPCR

Cells were lysed with Trizol (Invitrogen) and phase-separated with chloroform by centrifuging at 12,000 g for 15′ at 4 °C. RNA was isolated using the RNeasy kit (Qiagen), following manufacturer’s guidelines. cDNA generation was with Superscript VILO (Invitrogen), following manufacturer’s guidelines. LINC01013ORF was amplified by primer pair: 5′ GCTGCCCCTAAACGCATTCT 3′ (forward) and 5′ GGGCTT TTG GTG GGGATTCTT 3′ (reverse) in SYBR green real-time PCR master mix. Markers of fibroblast activation were measured by Taqman probes: ACTA2: Hs00909449_m1; POSTN: Hs01566734_m1; COL1A1: Hs00164004_m1; IL11: Hs01055413_g1; MMP2: Hs01548727_m1, FN1: Hs01549976_m1 and GUSB endogenous control: Hs99999908_m1, analysed with TaqMan Fast Advanced Master Mix (Thermofisher). Quantative PCR was performed on a QuantStudio 7 Flex Real-Time PCR System. Fold changes in gene expression were calculated using the 2(-Delta Delta C(T)) method [37].

### LINC01013ORF3 Overexpression

A codon optimised sequence with identical coding potential but altered RNA sequence to LINC01013ORF was cloned into expression vector pcDNA3.1 (Addgene) and expressed under control of a CMV promoter/enhancer. Control vector contained eGFP also expressed from CMV promoter/enhancer in the pMaxGFP vector (Lonza).

### Immunofluorescence

Immunofluorescence experiments were performed as described previously [25] with slight adjustments. HeLa cells were grown on 15 mm glass slides for 24 h and transfected with 3xFLAG tagged LINC01013ORF (LIN-C01013ORF-3xFLAG) in a pcDNA3.1 vector, using Lipofectamine 2000 reagent. Twenty-four-hour post transfection, cells were fixed with 4% paraformaldehyde for 10 min at room temperature and washed three times with ice-cold phosphate-buffered saline (PBS). Cells were permeabilized and blocked for 1 h at room temperature using 2.5% bovine albumin serum, 10% anti-goat serum and 0.1% Triton X or 30 mg/mL digitonin followed by 3 washing steps. Whilst Triton X permeabilizes the outer and inner mitochondrial membrane and therefore allows the staining of mitochondrial matrix proteins, digitonin leaves the inner mitochondrial membrane intact and prevents staining of mitochondrial matrix proteins. Overexpressed LINC01013ORF-3xFLAG was stained for 1 h at room temperature using an anti-FLAG mouse monoclonal antibody (1:500, F1804, Sigma Aldrich) and co-stained with the mitochondrial matrix protein ATPIF (1:1000, rabbit ATPIF1 #13,268, Cell Signaling Technology; Danvers, MA, USA) or the mitochondrial outer membrane protein TOM20 (1:200, rabbit TOM20 #42,406, Cell Signaling Technology; Danvers, MA, USA). Slides were then washed and incubated with fluorescently-labelled secondary antibodies (1:500, Alexa Fluor 488 anti-rabbit & Alexa Fluor 594 anti-mouse; Invitrogen, Carlsbad, CA, USA) for 30 min at room temperature. Cells were washed again, stained with 4–6-diamidino-2-phenylindole (NucBlue Fixed Cell ReadyProbes Reagent, R37606, Thermo Fisher) for 5 min at room temperature and mounted onto glass slides using ProLongTM Gold antifade reagent (Molecular Probes; InvitrogenTM). Images were visualised using a LEICA SP8 confocal microscope using a × 63 objective. Image analysis was performed using the Leica confocal software Las X (v3.5.2) and ImageJ (v1.52a).

### In Vitro *Translation of LINC01013 RNA*

The full LINC01013 RNA sequence (including 5′UTR, ORF region and 3′UTR) was synthesized and inserted into an expression plasmid by Genewiz Europe (Leipzig, Germany; construct available upon request). Linearized plasmid DNA (0.5 μg) was transcribed and translated in vitro as described previously [25] using the TnT Coupled Wheat Germ Extract system (Promega, Mannheim, Germany) in the presence of 10 mCi/mL [35S]-methionine (Hartmann Analytic, Braunschweig, Germany) according to manufacturer’s instructions. To visualise the translation products, 5μL lysate was denatured for 2 min at 85 °C in 9.6 μL Novex Tricine SDS Sample Buffer (2X) (Thermo Fisher Scientific) and 1.4 μL DTT (500 mM). Proteins were separated on 16% Tricine gels (Invitrogen) for 1 h at 50 V followed by 3.5 h at 100 V and blotted on PVDF-membranes (Immobilon-PSQ Membrane, Merck Millipore).

### Statistics

Continuous data were expressed as mean ± SEM. For two groups with normal distribution, data were compared using Student’s *t*-test with multiple test correction if required. For normally distributed data with more than two groups and one dependent variable, one-way ANOVA was used, with Tukey’s multiple test correction. For normally distributed data with more than two groups and two dependent variables, two-way ANOVA was used with Tukey’s multiple test correction. Nonparametric data of more than two groups was analysed using Friedmann’s test. Statistical analysis was performed with Prism 8.4.3.

## Results

### LINC01013 Is Expressed in Activated Human Cardiac Fibroblasts and Contains an Actively Translated smORF

**Fig. 1:**
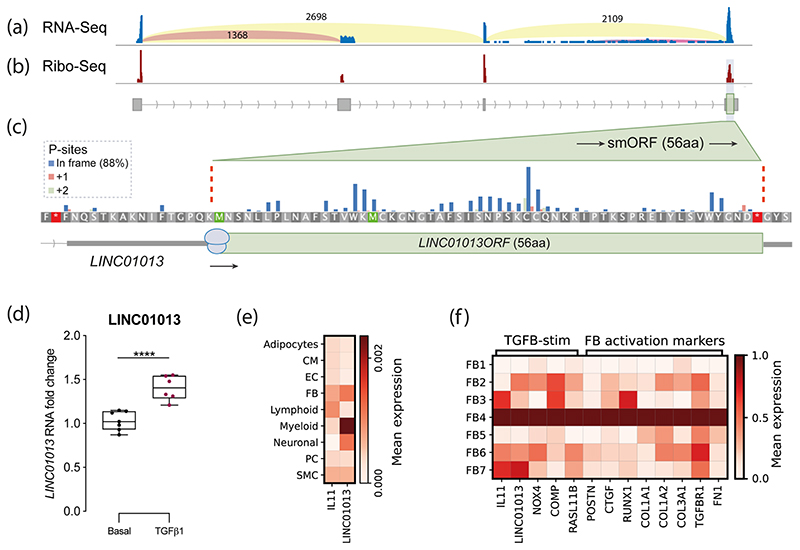
LINC01013 is expressed in cardiac fibroblasts and encodes a novel micropeptide: LINC01013ORF. **a** RNA-Seq sashimi plot of RNA transcripts derived from the *LINC01013* gene identified in human cardiac atrial fibroblasts. Light-blue vertical bars indicate RNA read depth within exons, and arced lines quantify reads across high-confidence exon-exon junctions. Two transcripts were identified corresponding to NR_038981.1 (e1 + e3 + e4; herein named LINC01013) and NR_146223.1 (e1 + e2). **b**, **c** Ribo-Seq analysis of identifies a high-confidence translated smORF within exon 4 contained in the LINC01013 transcript. Red bars in **b** show high densities of Ribo-Seq reads, of which are 88% in frame (blue bars, **c**) of a translated smORF: LINC01013ORF (green box, **c**). High ribosome drop-off at the LINC01013ORF stop codon further supports active translation of this ORF (data not shown). Arrowed lines: intron; grey boxes: exon, green: translated region. **d** LINC01013 expression in HCFs is increased by TGFβ1 stimulation in vitro, using primers amplifying LINC01013ORF. *N* = 6. *** = *p* < 0.0001. **e** Nuc-Seq data showing that LINC01013 is expressed in fibroblasts, myeloid and neuronal cells. CM: cardiomyocyte, EC: endothelial cell, FB: fibroblast, PC: pericyte, SMC: smooth muscle cell. **f** LINC01013 expression is associated with markers of TGFβ1 stimulation and fibroblast activation, and ECM-producing fibroblast subtype ‘FB4’

In order to establish the profile of LINC01013 RNA expression in cardiac fibroblasts, we first evaluated transcripts using data previously derived from primary human cardiac fibroblasts (HCFs) activated with TGFβ1 and analysed by RNA-Seq [8]. This identified two alternate transcripts: a major form corresponding to NR_038981.1 and a shorter, minor form corresponding to NR_146223.1 (Fig. 1a). We interrogated transcripts for potentially translated regions using ribosome profiling (Ribo-Seq) data likewise obtained from TGFβ1-stimulated HCFs [31]. This identified four smORFs: three of these had only limited evidence of translation but one, located in exon 4 of and within the major NR_038981.1 transcript, had a notably high density of ribosome footprints with consistent trinucleotide periodicity, highly indicative of active translation (Fig. 1b-c). This smORF spans 168 bp, which has an ATG in a moderate Kozak context (AAG**ATG** A), with coding potential for a 56AA micropeptide of a predicted mass of 6.3KDa. The NR_038981.1 transcript (hereon termed LINC01013) was therefore prioritised for further study together with the smORF contained therein (hereon named LINC01013ORF).

To evaluate LINC01013 expression in vivo, we interrogated single cell nucleus RNA sequencing (Nuc-Seq) data previously compiled from 14 healthy human hearts [38]. LINC01013 was detected almost exclusively in fibroblasts, myeloid cells and neuronal cells (Fig. 1e). Within the fibroblast population, LINC01013 was associated with an extracellular matrix-producing cluster of fibroblasts (described as ‘FB4’ in Litviňuková et al. [38]), which express a range of markers of TGFβ1 stimulation and fibroblast activation (Fig. 1f). Conversely, LINC01013 was not associated with the quiescent ‘FB1’ fibroblast subcluster, which has the lowest expression of TGFβ1- and fibroblast activation-associated genes. LINC01013 was expressed in fibroblasts across all regions of the healthy human heart (Supplementary Fig. 1; information on fibroblast subtypes can be found in Supplementary Table 1).

Upregulation of the LINC01013 transcript under the experimental conditions used here was confirmed using cultured primary HCFs analysed by qPCR. LINC01013 expression was readily detectable and consistently and robustly upregulated after treatment with 5 ng/ml TGFβ1 for 24 h (Fig. 1d).

### siRNA-Mediated Knockdown of LINC01013 Reduces the Fibrotic Phenotype at Baseline and After TGFβ1 Stimulation

To evaluate the effect of knocking down LINC01013 RNA on the fibrotic phenotype, we used a model of fibroblast activation: HCFs were serum-starved for 16 h, then treated with 5 ng/ml of TGFβ1 for 24 h, after which RNA markers of fibroblast activation were quantified: α-smooth muscle actin *(ACTA2),* interleukin 11 *(IL11),* periostin *(POSTN),* fibronectin *(FN1)* and collagen 1α1 *(COL1A1)* and were robustly increased as determined by qPCR, whilst matrix metalloproteinase *(MMP2)* remained unchanged (Supplementary Fig. 2).

**Fig. 2:**
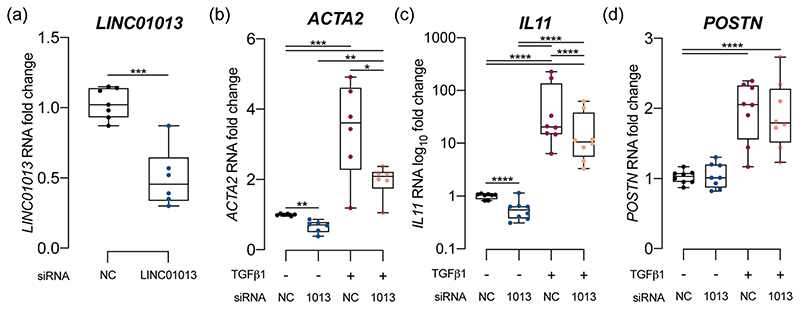
LINC01013 knockdown using siRNA reduces fibroblast activation and blunts the response to TGFB1. **a** LINC01013 RNA was knocked down effectively by anti-LINC01013 siRNA. **b**–**d** siRNA-mediated LINC01013 knockdown reduces markers of fibroblast activation *ACTA2* and *IL11* at baseline, and blunts their response to TGFβ1 stimulation. *POSTN* expression was not affected by LINC01013 knockdown. *N* = 6. * = *p* < 0.05, ** = *p* < 0.01, *** = *p* < 0.001, **** = *p* < 0.0001. NC: nontargeting control siRNA

We first assessed the effect of siRNA-mediated LINC01013 knockdown at baseline: HCFs were serum- starved for 16 h, before treatment with either anti- LINC01013 siRNA or nontargeting control (NC) siRNA. qPCR demonstrated effective knockdown of the endogenous LINC01013 RNA (Fig. 2a) and reduced fibroblast activation markers *ACTA2, IL11* (Fig. 2b, c) and *MMP2* (Supplementary Fig. 3) but not *POSTN* (Fig. 2d) at 24 h.

To evaluate the effect of LINC01013 knockdown on TGFβ1-mediated fibroblast activation, HCFs were serum starved for 16 h before treatment with either anti- LINC01013 siRNA or nontargeting control siRNA for 24 h and further cultured in either standard media or standard media + 5 ng/ml TGFβ1. LINC01013 knockdown blunted the response to TGFβ1, reducing *ACTA2* and *IL11* (Fig. 2b, c), but not *POSTN* (Fig. 2d) expression.

### Overexpression of the LINC01013ORF Leads to Fibroblast Activation

**Fig. 3:**
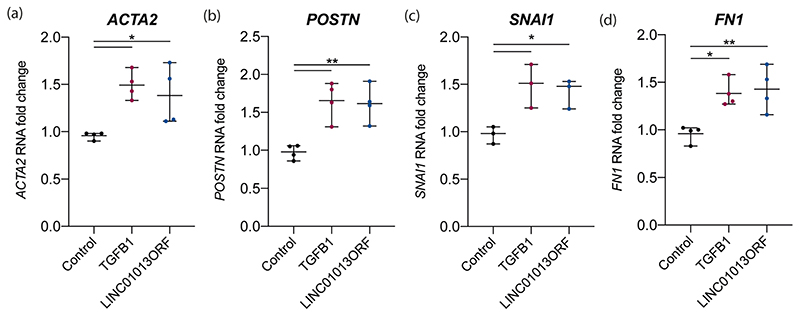
Overexpression of LINC1013ORF activates fibroblasts. **a**–**d** HCFs were transfected with either eGFP (control) or codon-optimised LINC01013ORF expression constructs. Cells transfected with control constructs were further treated with 5 ng/ml TGFβ1 for 24 h, whilst LINC01013ORF-transfected cells were cultured in media alone. TGFβ1 increased expression of *ACTA2*, *POSTN*, *SNAI1* and *FN1* in control cells. In the absence of TGFβ1, LINC01013ORF expression increased expression of *ACTA2*, *POSTN, SNAI1* and *FN1* to the same level as TGFβ1 stimulation. *N* = 4, * = *p* < 0.05, ** = *p* < 0.01, *** = *p* < 0.001

We first used in vitro translation to validate production of the LINC01013ORF protein product (Supplementary Fig. 4). To evaluate its functional role and to distinguish peptide driven effects, we used a codon-optimised form of LINC01013ORF, cloned into a pcDNA3.1 vector (pcDNA3.1-LINC01013ORF), driven by a constitutive CMV promoter. Control HCFs were transfected with an eGFP expression construct and transfection validated by visualisation of eGFP (Supplementary Fig. 5). In control cells, TGFβ1 treatment increased myofibroblast transdifferentiation markers: *ACTA2* and *POSTN*, profibrotic transcription factor *SNAI1* and its target ECM protein, *FN1.* Overexpression of LINC01013ORF increased these same markers (Fig. 3a-d) to the same level as seen with TGFβ1.

### LINC01013ORF Encodes a Micropeptide that Localises to the Mitochondrial Matrix

**Fig. 4:**
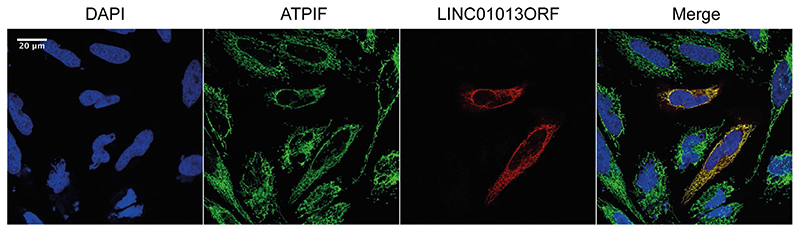
LINC01013ORF localises to the mitochondrial matrix. LINC01013-ORF-3 × FLAG was expressed in HeLa cells. Permeabilisation with triton-X and co-staining the mitochondrial matrix protein ATPIF (GFP) and LINC010FLAG (mCherry) demonstrated co-localisation of LINC01013ORFpep and mitochondria. *N* = 4, representative images are shown

To investigate the subcellular localisation of LIN-C01013ORF peptide, we overexpressed a LIN-C01013ORF-3 × FLAG fusion peptide in HeLa cells, and co-stained with DAPI (nuclei), anti-FLAG (LIN- C01013ORF-FLAG) and anti-ATPIF (mitochondrial matrix). When cells were permeabilised with triton-X, the peptide encoded by LINC01013ORF (LINC01013ORF-pep) clearly co-localised with ATPIF (Fig. 4) indicating its mitochondrial localisation. Additionally, we permea- bilised cells with digitonin which does not penetrate the mitochondrial inner membrane and thus prevents antibody staining of mitochondrial matrix proteins, but permits that of those in the intermembrane space or outer mitochondrial membrane (OMM). Dropout of both ATPIF and FLAG signals, but not TOMM20 (OMM protein) with digitonin permeabilisation (Supplementary Fig. 6), indicates that LINC01013ORF micropeptide, like ATPIF, localises to the mitochondrial matrix.

## Discussion

We have demonstrated that LINC01013 is upregulated in the response of cardiac fibroblasts to TGFβ1, and that it harbours a previously undocumented smORF that is translated and encodes a biologically active peptide. Knockdown of LINC01013 reduces markers of fibroblast activation at baseline and blunts the response to TGFβ1, whilst overexpression of LINC01013ORF induces markers of activation to the same level as seen with TGFβ1 stimulation. To our knowledge, LINC01013ORF is the only known translated smORF described to date to be associated with TGFβ1 signalling.

To date LINC01013 has remained largely uncharacterised and the presence of a smORF encoded peptide has not been previously reported. Our data show that LINC01013 is directly implicated in TGFβ1-mediated fibroblast activation and that this effect is mediated, at least in part, due to the presence of a smORF encoded peptide. Whilst this study was under review, others have reported similar activation of LINC01013 by TGFβ1 in aortic valve fibroblasts [39].

TGFβ1-mediated fibroblast activation can occur both via canonical (SMAD-mediated) and noncanonical (SMAD- independent) pathways [40]. We propose that the effects of LINC01013ORF peptide may act primarily through the canonical pathway: siRNA-LINC01013 mediated knockdown reduced expression of SMAD-dependent genes *ACTA2* and *IL11,* but did not reduce *POSTN* (Fig. 2) the expression of which is upregulated by noncanonical p38 MAPK [41]. LINC01013ORF overexpression activated the canonical target genes *ACTA2* and *SNAI1* (Fig. 3). It also resulted in increased *FN1* and *POSTN* expression which is likely due to direct *SNAI1*-mediated transcription [42], or activation of the Wnt/β-catenin, pathway which is known to act on a broad range of genes involved in fibroblast activation [43]. Our data therefore align with previous findings of a LINC01013-SNAIL1-FN1 pathway [27], and suggest the translated ORF within LINC01013 may be the active player involved.

Further supportive of its role in TGFβ1 signalling, we show that in the human heart, LINC01013 is positively associated with the previously described [38] subpopulation of fibroblasts that express markers of TGFβ1 activation and ECM production, and negatively associated with a subpopulation of quiescent fibroblasts.

The mechanisms by which LINC01013ORF encoded peptide acts upon TGFβ1 signalling remain uncertain. When overexpressed, LINC01013ORF peptide localises to the mitochondria. Mitochondria-mediated metabolic drivers that broadly activate fibroblasts are well described, acting via reactive oxygen species (ROS) activating p38 and ERK1/2 pathways [44]. It is therefore possible that the profibrotic effect of LINC01013ORF peptide may be through overall metabolic stress. Of note, overexpression can influence a protein localisation and future work should resolve the localisation of the endogenous protein to substantiate the connection to mitochondria and fibroblast activation. FLAG-tag fusion can also potentially influence subcellular localisation, though has not typically induced mitochondrial localisation in the study of other smORFs [25].

More broadly, our data demonstrate how Ribo-Seq can be leveraged in the discovery of novel translated targets. Many lncRNAs have been implicated in cardiac disease [45], including myocyte dysfunction [46] and as potential biomarkers post infarct [47], and our work highlights the importance of considering an active role of biologically active translated smORFs within these: there is a growing body of evidence for functional smORF peptides [22, 23, 48]. lncRNAs harbouring smORFs may thus potentially act via coding and non-coding mechanisms, with synergistic or independent functions.

In conclusion, we propose that LINC01013 functions downstream of TGFβ1 and may represent a potential therapeutic target in limiting fibroblast activation. We have demonstrated it to be both necessary and sufficient for fibroblast activation at the RNA level, acting potentially via the canonical TGFβ1 pathway. Fibrosis is a determinant of clinical outcome in the heart, liver, lung and kidneys, and so large cohorts of patients with established fibrosis, or who are at risk of its development, can potentially benefit from antifibrotic therapy. To our knowledge, LINC01013ORF is the first smORF to be directly implicated in fibroblast activation, and our work highlights the importance to consider biologically active smORFs within putatively non-coding regions. Finally, whilst our observations have been made in cardiac fibroblasts, due to mechanistic homology, they are potentially translatable to fibrosis in other tissues.

## Supplementary Material

The online version contains supplementary material available at https://doi.org/10.1007/s12265-022-10288-z.

Supplementary Materials

## Abbreviations

CMV: Cytomegalovirus
DMEM: Dulbecco’s modified eagle medium
ECM: Extracellular matrix
HCF: Human cardiac fibroblast
HEK: Human embryonic kidney
HUVEC: Human umbilical cord vascular endothelial cell
lincRNA: Long intergenic non-coding ribonucleic acid
lncRNA: Long non-coding ribonucleic acid
Nuc-Seq: Nuclear RNA sequencing
ORF: Open reading frame
RNA: Ribonucleic acid
RNA-Seq: Ribonucleic acid sequencing
RPF: Ribosome-protected fragment
siRNA: Small-interfering ribonucleic acid
smORF: Small open reading frame
STAR: Spliced transcript alignment to a reference
TGFβl: Transforming growth factor beta 1
UTR: Untranslated region

## References

[1] Gulati A, Japp AG, Raza S, Halliday BP, Jones DA, Newsome S, Prasad SK (2018). Absence of myocardial fibrosis predicts favorable long-term survival in new-onset heart failure. Circulation: Cardiovascular Imaging.

[2] Burstein B, Nattel S (2008). Atrial fibrosis: Mechanisms and clinical relevance in atrial fibrillation. Journal of the American College of Cardiology.

[3] Rockey DC, Bell PD, Hill JA (2015). Fibrosis — A common pathway to organ injury and failure. New England Journal of Medicine.

[4] Heymans S, González A, Pizard A, Papageorgiou AP, Lõpez-Andrés N, Jaisser F, Díez J (2015). Searching for new mechanisms of myocardial fibrosis with diagnostic and/or therapeutic potential. European Journal of Heart Failure.

[5] Shull MM, Ormsby I, Kier AB, Pawlowski S, Diebold RJ, Yin M, Doetschman T (1992). Targeted disruption of the mouse transforming growth factor-β1 gene results in multifocal inflammatory disease. Nature.

[6] Bierie B, Chung CH, Parker JS, Stover DG, Cheng N, Chytil A, Moses HL (2009). Abrogation of TGF-β signaling enhances chemokine production and correlates with prognosis in human breast cancer. Journal of Clinical Investigation.

[7] Tallquist MD, Molkentin JD (2017). Redefining the identity of cardiac fibroblasts. Nature Reviews Cardiology.

[8] Schafer S, Viswanathan S, Widjaja AA, Lim WW, Moreno-Moral A, DeLaughter DM, Cook SA (2017). IL11 is a crucial determinant of cardiovascular fibrosis. Nature.

[9] Batlle R, Alba-Castellón L, Loubat-Casanovas J, Armenteros E, Francí C, Stanisavljevic J, De Herreros AG (2013). Snail1 controls TGF-β responsiveness and differentiation of mesenchymal stem cells. Oncogene.

[10] Biswas H, Longmore GD (2016). Action of SNAIL1 in cardiac myofibroblasts is important for cardiac fibrosis following hypoxic injury. PLoS ONE.

[11] Horiguchi K, Shirakihara T, Nakano A, Imamura T, Miyazono K, Saitoh M (2009). Role of Ras signaling in the induction of snail by transforming growth factor-β. Journal of Biological Chemistry.

[12] Baulida J, Díaz VM, García de Herreros A (2019). Snail1: A transcriptional factor controlled at multiple levels. Journal of Clinical Medicine.

[13] Boutet A, De Frutos CA, Maxwell PH, Mayol MJ, Romero J, Nieto MA (2006). Snail activation disrupts tissue homeostasis and induces fibrosis in the adult kidney. EMBO Journal.

[14] Kanisicak O, Khalil H, Ivey MJ, Karch J, Maliken BD, Correll RN, Molkentin JD (2016). Genetic lineage tracing defines myofibroblast origin and function in the injured heart. Nature Communications.

[15] Chen J, Brunner AD, Cogan JZ, Nuñez JK, Fields AP, Adamson B, Weissman JS (2020). Pervasive functional translation of noncanonical human open reading frames. Science.

[16] Couso JP, Patraquim P (2017). Classification and function of small open reading frames. Nature Reviews Molecular Cell Biology.

[17] Saghatelian A, Couso JP (2015). Discovery and characterization of smORF-encoded bioactive polypeptides. Nature Chemical Biology.

[18] Ingolia NT, Brar GA, Stern-Ginossar N, Harris MS, Tal-houarne GJS, Jackson SE, Weissman JS (2014). Ribosome profiling reveals pervasive translation outside of annotated protein-coding genes. Cell Reports.

[19] Ingolia NT, Ghaemmaghami S, Newman JRS, Weissman JS (2009). Genome-wide analysis in vivo of translation with nucleotide resolution using ribosome profiling. Science.

[20] Ingolia NT, Brar GA, Rouskin S, McGeachy AM, Weissman JS (2012). The ribosome profiling strategy for monitoring translation in vivo by deep sequencing of ribosome-protected mRNA fragments. Nature Protocols.

[21] Makarewich CA, Munir AZ, Schiattarella GG, Bezprozvannaya S, Raguimova ON, Cho EE, Olson EN (2018). The DWORF micropeptide enhances contractility and prevents heart failure in a mouse model of dilated cardiomyopathy. eLife.

[22] Nelson BR, Makarewich CA, Anderson DM, Winders BR, Troupes CD, Wu F, Olson EN (2016). A peptide encoded by a transcript annotated as long noncoding RNA enhances SERCA activity in muscle. Science.

[23] Anderson DM, Anderson KM, Chang CL, Makarewich CA, Nelson BR, McAnally JR, Olson EN (2015). A micropeptide encoded by a putative long noncoding RNA regulates muscle performance. Cell.

[24] Makarewich CA, Bezprozvannaya S, Gibson AM, Bassel-Duby R, Olson EN (2020). Gene therapy with the DWORF micropeptide attenuates cardiomyopathy in mice. Circulation Research.

[25] van Heesch S, Witte F, Schneider-Lunitz V, Schulz JF, Adami E, Faber AB, Hubner N (2019). The translational landscape of the human heart. Cell.

[26] Yang H, Cao Y, Zhang J, Liang Y, Su X, Zhang C, Fan Z (2020). DLX5 and HOXC8 enhance the chondrogenic differentiation potential of stem cells from apical papilla via LINC01013. Stem Cell Research and Therapy.

[27] Chung IH, Lu PH, Lin YH, Tsai MM, Lin YW, Yeh CT, Lin KH (2017). The long non-coding RNA LINC01013 enhances invasion of human anaplastic large-cell lymphoma. Scientific Reports.

[28] Wang W, Xu S, Di Y, Zhang Z, Li Q, Guo K, Wang B (2021). Novel role of LINC01013/miR-6795-5p/FMNL3 axis in the regulation of hepatocellular carcinoma stem cell features. Acta biochimica et biophysica Sinica.

[29] Pham TP, Bink DI, Stanicek L, van Bergen A, van Leeu-wen E, Tran Y, Boon RA (2021). Long non-coding RNA aer-rie controls DNA damage repair via YBX1 to maintain endothelial cell function. Frontiers in Cell and Developmental Biology.

[30] Chandra S, Ehrlich KC, Lacey M, Baribault C, Ehrlich M (2021). Epigenetics and expression of key genes associated with cardiac fibrosis: NLRP3, MMP2, MMP9, CCN2/CTGF, and AGT. Epigenomics.

[31] Chothani S, Schäfer S, Adami E, Viswanathan S, Widjaja AA, Langley SR, Rackham OJL (2019). Widespread translational control of fibrosis in the human heart by RNA-binding proteins. Circulation.

[32] Bolger AM, Lohse M, Usadel B (2014). Trimmomatic: A flexible trimmer for Illumina sequence data. Bioinformatics.

[33] Langmead B, Trapnell C, Pop M, Salzberg SL (2009). Ultrafast and memory-efficient alignment of short DNA sequences to the human genome. Genome Biology.

[34] Dobin A, Davis CA, Schlesinger F, Drenkow J, Zaleski C, Jha S, Gingeras TR (2013). STAR: Ultrafast universal RNA-seq aligner. Bioinformatics.

[35] Liao Y, Smyth GK, Shi W (2013). The Subread aligner: Fast, accurate and scalable read mapping by seed-and-vote. Nucleic Acids Research.

[36] Xiao Z, Huang R, Xing X, Chen Y, Deng H, Yang X (2018). De novo annotation and characterization of the translatome with ribosome profiling data. Nucleic Acids Research.

[37] Livak KJ, Schmittgen TD (2001). Analysis of relative gene expression data using real-time quantitative PCR and the 2(-Delta Delta C(T)) method. Methods.

[38] Litviňuková M, Talavera-López C, Maatz H, Reichart D, Worth CL, Lindberg EL, Teichmann SA (2020). Cells of the adult human heart. Nature.

[39] Chignon A, Argaud D, Boulanger M-C, Mkannez G, Bon-Baret V, Li Z, Mathieu P (2022). Genome-wide chromatin contacts of super-enhancer-associated lncRNA identify LINC01013 as a regulator of fibrosis in the aortic valve. PLOS Genetics.

[40] Meng XM, Nikolic-Paterson DJ, Lan HY (2016). TGF-β: The master regulator of fibrosis. Nature Reviews Nephrology.

[41] Li L, Fan D, Wang C, Wang JY, Cui XB, Wu D, Wu LL (2011). Angiotensin II increases periostin expression via Ras/p38 MAPK/CREB and ERK1/2/TGF-β1 pathways in cardiac fibroblasts. Cardiovascular Research.

[42] Cano A, Pérez-Moreno MA, Rodrigo I, Locascio A, Blanco MJ, Del Barrio MG, Nieto MA (2000). The transcription factor snail controls epithelial-mesenchymal transitions by repressing E-cadherin expression. Nature Cell Biology.

[43] Akhmetshina A, Palumbo K, Dees C, Bergmann C, Venalis P, Zerr P, Distler JHW (2012). Activation of canonical Wnt signalling is required for TGF-β-mediated fibrosis. Nature Communications.

[44] Gibb AA, Lazaropoulos MP, Elrod JW (2020). Myofibroblasts and fibrosis: Mitochondrial and metabolic control of cellular differentiation. Circulation Research.

[45] Statello L, Guo CJ, Chen LL, Huarte M (2021). Gene regulation by long non-coding RNAs and its biological functions. Nature Reviews Molecular Cell Biology.

[46] Sun J, Wang R, Chao T, Wang C (2021). Long noncoding RNAs involved in cardiomyocyte apoptosis triggered by different stressors. Journal of Cardiovascular Translational Research.

[47] Yan L, Zhang Y, Zhang W, Deng SQ, Ge ZR (2020). lncRNA-NRF is a potential biomarker of heart failure after acute myocardial infarction. Journal of Cardiovascular Translational Research.

[48] Anderson DM, Makarewich CA, Anderson KM, Shelton JM, Bezprozvannaya S, Bassel-Duby R, Olson EN (2016). Widespread control of calcium signaling by a family of SERCA-inhibiting micropeptides. Science Signaling.

