# Correction to: LINC01013 Is a Determinant of Fibroblast Activation and Encodes a Novel Fibroblast-Activating Micropeptide

**DOI:** 10.1007/s12265-022-10291-4

**Published:** 2022-07-14

**Authors:** N. M. Quaife, S. Chothani, J. F. Schulz, E. L. Lindberg, K. Vanezis, E. Adami, K. O’Fee, J. Greiner, M. Litviňuková, S. van Heesch, N. Whiffin, N. Hubner, S. Schafer, O. Rackham, S. A. Cook, P. J. R. Barton

**Affiliations:** 1 National Heart and Lung Institute, Imperial College London, London, UK; 2 MRC London Institute of Medical Sciences, London, UK; 3 Program in Cardiovascular and Metabolic Disorders, Duke-National University of Singapore, Singapore, 169857 Singapore; 4 Max-Delbrück-Center for Molecular Medicine in the Helmholtz Association (MDC), Berlin, Germany; 5 DZHK (German Center for Cardiovascular Research), partner site Berlin, Berlin, Germany; 6 Princess Máxima Center for Pediatric Oncology, Utrecht, The Netherlands; 7 Cardiovascular Research Centre, Royal Brompton and Harefield Hospitals, Guy’s and St Thomas NHS Foundation Trust, London, UK; 8 Wellcome Centre for Human Genetics, University of Oxford, Oxford, UK; 9 Berlin Institute of Health at Charité – Universitätsmedizin Berlin, Berlin, Germany; 10 National Heart Centre Singapore, Singapore, Singapore

**Correction to: Journal of Cardiovascular Translational Research**

**https://doi.org/10.1007/s12265-022-10288-z**

Due to an error during production, the graphical abstract for this article was missing from the article as originally published.

The original article has been corrected.

