# Supplementary material for: LINC01013 Is a Determinant of Fibroblast Activation and Encodes a Novel Fibroblast-Activating Micropeptide
Source: J Cardiovasc Transl Res. 2022 Jun 27;16(1):77–85. doi: 10.1007/s12265-022-10288-z (PMC9944705; doi:10.1007/s12265-022-10288-z)
Supplement: Supplementary file 1 [file 12265_2022_10288_MOESM1_ESM.docx]

Supplementary material

**Supplementary Table 1. Fibroblast states as described in Litviňuková** ***et al.*****, 2020** [39].

| **Subtype** | **Characteristics** | **Marker genes** |
| --- | --- | --- |
| FB1 | Ground state, ventricular | *SCN7A, BMPER, ACSM1* |
| FB2 | Ground state, atrial | *CFH, ID4, KCNT2* |
| FB3 | MP-interacting | *PTX3, OSMR, IL6ST* |
| FB4 | TGFβ1 activated | *POSTN, TNC, FAP* |
| FB5 | ECM organising/remodelling | *FBLN2, PCOLE2, LINC01133* |
| FB6 | Stromal | *CD36, EGFLAM, FTL1* |

**Supplementary Fig. 1. LINC01013 is expressed in all regions of healthy human hearts**

Transmural biopsies were obtained from healthy human hearts (as described in Litviňuková *et al.*, 2020 [39]) from the following regions: AX: left ventricular apical core, LA: left atrium, LV: left ventricle, RA: right atrium, RV: right ventricle, SP: septum. Nuc-Seq was performed, and library size-adjusted, log-transformed counts of LINC01013 in the fibroblast population of each region are presented. LINC01013 is expressed the highest in LA and RV fibroblasts, with appreciable quantities in all chambers. 

**Supplementary Fig.2. TGF**β**1 stimulation elicits a fibrotic response in HCFs**

HCFs were cultured in either media alone, or media containing 5ng/ml TGFβ1. After 24h RNA was extracted and analysed for markers of fibroblast activation and fibrosis. TGFβ1 treatment elicited an increase in *ACTA2, IL11, POSTN, COL1A1* and *FN1* RNAs. *MMP2* RNA was not increased. N = 6. *: p < 0.05, **: p < 0.01, 

***: p < 0.001, ****: p < 0.0001. 

**Supplementary Fig. 3. siRNA-mediated knockdown decreases** ***MMP2*** **RNA in HCFs**

HCFs were transfected with either nontargeting control siRNA (NC) or anti-LINC01013 siRNA. After 24h, RNA was extracted and analysed for markers of fibroblast activation and fibrosis as described in Fig. 2. Treatment with anti-LINC01013 siRNA decreased *MMP2* RNA. *=p<0.05 (dataset also includes Fig. 2a-d).

**Supplementary Fig. 4. LINC01013ORF peptide can be expressed and detected** ***in vitro***

Full-length LINC01013 was cloned into pcDNA3.1 expression vector and expressed *in vitro* in the presence of [35S]-methionine. Incorporation of [35S]-methionine into newly synthesized proteins enabled the detection of translation products by phosphor imaging (exposure time 1d). A band corresponding to LINC01013ORF micropeptide was detected at the expected size of 6.3kDa alongside molecular weight markers: MP3: 10.9Kb, MP4: 3.1Kb, MP5: 5.2Kb and MP6: 5.1Kb

**Supplementary Fig. 5. Human cardiac fibroblasts transfected with pMaxGFP expression vector**

Human cardiac fibroblasts were Nucleofected, as described in Methods, with 2.5mg pMaxGFP expression vector per 750,000 cells. Efficacy of transfection was validated by visualisation using a Leica DMi8 inverted microscope.

**Supplementary Fig. 6. Permeabilisation of HeLa cells with digitonin does not permit staining of LINC01013 peptide, which localises to the mitochondrial matrix**

(a) permeabilisation of HCFs with digitonin (which does not permeabilise the mitochondrial inner membrane) allows nuclear staining (DAPI) but dropout of ATPIF and LINC01013ORF-3xFLAG signals compared to permeabilization with TritonX. This suggests that ATPIF and LINC01013ORF localise to the intermembrane space. (b) permeabilisation with digitonin permits control immunostaining of TOM20, an external membrane protein. N=4. Illustrative images are shown.
